# Auditory motion-specific mechanisms in the primate brain

**DOI:** 10.1371/journal.pbio.2001379

**Published:** 2017-05-04

**Authors:** Colline Poirier, Simon Baumann, Pradeep Dheerendra, Olivier Joly, David Hunter, Fabien Balezeau, Li Sun, Adrian Rees, Christopher I. Petkov, Alexander Thiele, Timothy D. Griffiths

**Affiliations:** Institute of Neuroscience, Newcastle University, Newcastle upon Tyne, Tyne and Wear, United Kingdom; Cold Spring Harbor Laboratory, United States of America

## Abstract

This work examined the mechanisms underlying auditory motion processing in the auditory cortex of awake monkeys using functional magnetic resonance imaging (fMRI). We tested to what extent auditory motion analysis can be explained by the linear combination of static spatial mechanisms, spectrotemporal processes, and their interaction. We found that the posterior auditory cortex, including A1 and the surrounding caudal belt and parabelt, is involved in auditory motion analysis. Static spatial and spectrotemporal processes were able to fully explain motion-induced activation in most parts of the auditory cortex, including A1, but not in circumscribed regions of the posterior belt and parabelt cortex. We show that in these regions motion-specific processes contribute to the activation, providing the first demonstration that auditory motion is not simply deduced from changes in static spatial location. These results demonstrate that parallel mechanisms for motion and static spatial analysis coexist within the auditory dorsal stream.

## Author summary

Motion is a fundamental dimension of acoustic and visual stimuli that is critical for animals to interact with their environment. Yet, surprisingly, we still do not understand the basic mechanisms in the brain that underlie perception of auditory motion. For the last 30 y, this research field has been hampered by unsuccessful attempts to answer a simple but fundamental question: is auditory motion perception deduced from processing individual static sounds, or are there mechanisms in the auditory domain dedicated to detecting motion? Here we report the discovery of specific motion detectors located in the auditory cortex of primates. We demonstrate that these auditory motion detectors are close to the well-known visual motion detectors. Both types of detectors are likely to be crucial for the planning of limb and eye movement. This study addresses a fundamental issue in neuroscience and sheds new light on the brain mechanisms underlying the essential aspects of our ability to navigate the world.

## Introduction

Motion is a fundamental dimension of acoustic and visual stimuli that is critical for animals to interact with their environment. Human psychoacoustic studies have addressed whether auditory motion analysis depends on sequential perception of stationary sources or whether specific motion detection mechanisms exist, but the results so far have remained inconclusive [1–9].

Studies of neuronal activity in various mammalian species, including macaques, have shown that cues supporting auditory motion perception can induce pronounced asymmetry of neuronal responses to opposite motion directions in the inferior colliculus and primary auditory cortex A1 [10,11]. However, it has been argued that this apparent direction sensitivity does not represent genuine motion selectivity but results from “adaptation of excitation,” defined as the reduced capacity of a neuron to respond to excitatory stimuli following the presentation of a prior excitatory stimulus, a mechanism also called “spatial masking” [12–14]. Motion processing per se has not been investigated beyond A1 in animal models. However, sensitivity to static spatial information has been shown to increase from A1 to the caudomedial (CM) and caudolateral (CL) belt areas in macaques [15–19], opening the possibility that these regions might be more sensitive to dynamic spatial information than A1.

Human functional magnetic resonance imaging (fMRI) studies indicate that the planum temporale, the region of the auditory cortex that contains areas homologous to monkey areas CM and CL, is involved in auditory motion processing [20,21]. However, these studies did not directly address the question of the underlying mechanisms and did not allow for any conclusion about the existence of specific motion-detection processes for sound movement analysis as opposed to sequential processing of stationary sources. Similarly, lesion studies did not allow for distinguishing between the two hypotheses [22–25].

In this study, we measured the fMRI blood-oxygen-level-dependent (BOLD) response to auditory motion in the whole auditory cortex of awake macaques. We performed a series of experiments designed to elucidate the mechanisms supporting auditory motion processing. Data revealed that auditory motion perception relies on specific computational mechanisms beyond the simple representation of successive snapshots of location.

## Results

**Fig 1:**
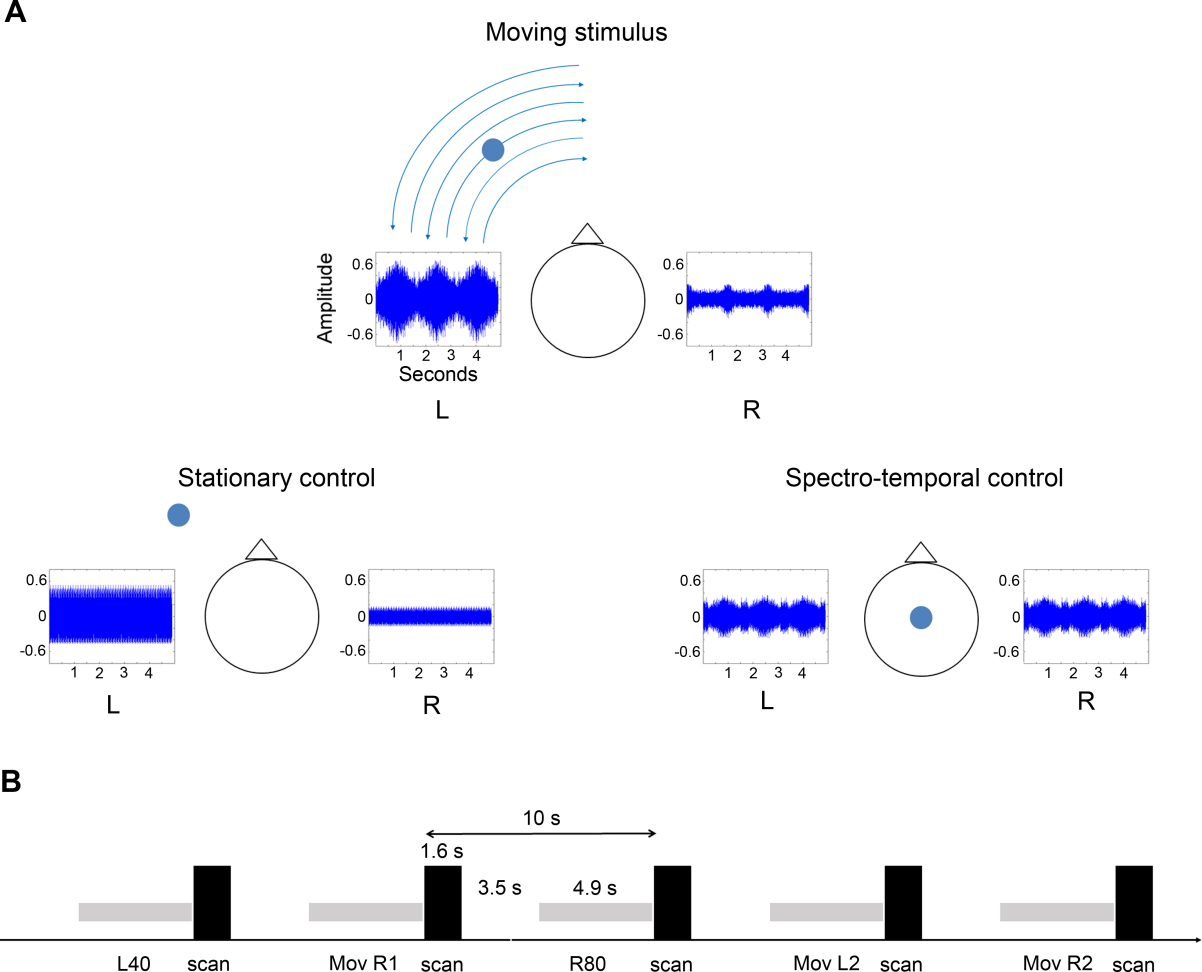
Stimuli and experimental paradigm. (A) Representation of different exemplars of the auditory stimuli used in experiments 1 to 4. The plots represent the waveform of one moving stimulus, one stationary control stimulus, and one spectrotemporal stimulus. The pictograms represent the location of each stimulus relative to the subject’s head, as perceived during the experiments. L: left, R: right. (B) Schematic representation of the main experimental paradigm. We used a sparse-sampling paradigm where 4.9 s-long auditory stimuli were presented between each scan acquisition (every 10 s). The presentation order of the stimuli was pseudorandomized. Stimuli illustrated: L40: stationary stimulus coming from the −40° position; Mov R1: moving stimulus moving within the right hemispace, starting from the 0° position; R80: stationary stimulus coming from the +80° position; Mov L2: moving stimulus moving within the left hemispace, starting from the −80° position; and Mov R2: moving stimulus moving within the right hemispace, starting from the +80° position.

We employed virtual auditory motion stimuli smoothly moving in azimuth at a speed of 100°/s and different control stimuli (Fig 1A). The choice of the movement plane and the speed of the motion stimuli were chosen to be ecologically relevant (consistent with everyday listening experience) and to take advantage of the fact that human and macaque listeners are more accurate in perceiving angular sound position change in the azimuthal plane than in the vertical one [17,26,27].

### Experiments 1 and 2: Motion versus stationary stimuli

The two first experiments involved motion and stationary stimuli. Four moving and five stationary stimuli were presented in a random order to subjects involved in a visual fixation task, and a sparse-sampling paradigm was used to measure the BOLD response induced by each stimulus (Fig 1B). Auditory stimuli were based on individual intra-auricular recordings of an amplitude-modulated broadband noise. Virtual moving stimuli were perceived as moving back and forth between the positions 0° and 80° within one hemispace, with half of the stimuli starting from the midline position (0°), while the other half started from the most lateral position (80°), resulting in four motion stimulus exemplars per subject. The five stationary stimuli corresponded to sounds perceived as coming from the locations −80°, −40°, 0, +40°, and +80°, respectively (the minus sign referring to the left hemispace and the plus sign to the right one). Because moving stimuli starting from the central position induced similar BOLD responses as those starting to move from the periphery, data were pooled, resulting in two motion conditions corresponding to sounds moving within the left hemispace (*Motion Left*) and those moving within the right hemispace (*Motion Right*).

The goal of this experiment was to identify the neural substrates of motion perception that were not explained by the processing of stationary sounds. We thus compared the BOLD response induced by moving sounds within each hemispace with the average BOLD response induced by the stationary stimuli corresponding to the spatial positions through which the moving sounds passed: the BOLD responses induced by stationary stimuli −80°, −40°, and 0° were thus averaged and compared to those induced by sounds moving in the left hemispace (contrast *Motion Left minus Stationary Left*), while the BOLD responses induced by stationary stimuli +80°, +40°, and 0° were averaged and compared to those induced by sounds moving in the right hemispace (contrast *Motion Right minus Stationary Right*). These comparisons allowed us to control for the laterality of the motion stimuli (nondynamic spatial information, corresponding to the encoding of space within which the motion stimulus was moving), as well as for their intrinsic spectrotemporal content (amplitude-modulated broadband noise filtered through the pinna).

**Fig 2:**
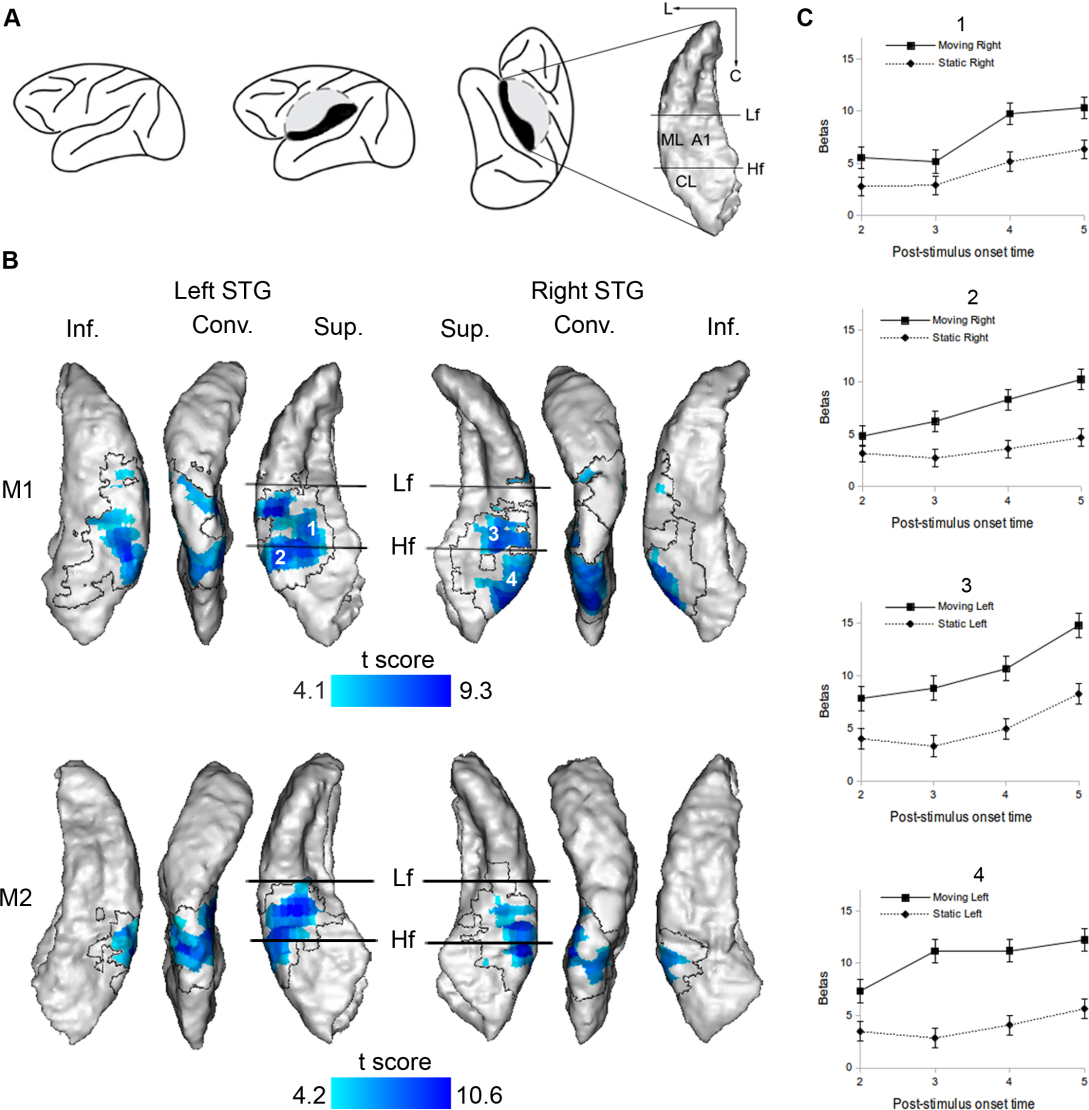
*Motion minus Stationary* contrast. (A) Location of the superior temporal gyrus (STG) surface and of the auditory areas in context. The dashed line corresponds to a cut through the operculum to expose the superior bank of the STG (in black). The relative positions of the primary core area, A1, and the secondary belts areas, middle lateral (ML) and caudolateral (CL), are indicated on the STG surface. The two straight lines represent the low-frequency (Lf) and high-frequency (Hf) reversals of tonotopy gradients. L; lateral; C: caudal. See also Materials and Methods and S1 Fig. (B) *Motion minus Stationary* contrasts (Experiment 1). Blood-oxygen-level-dependent (BOLD) responses were measured 5 s after the stimulus onset. Significant statistical parametric *t* maps are shown on a surface rendering of the STG of each individual (number of scans [*n*] = 2,466 [monkey 1, M1] and 3,429 [monkey 2, M2]; degrees of freedom [df] = 2,210 [M1] and 3,071 [M2]; *p*-values range after correction with the family-wise error (FWE) method [P_FWE_] from 0.05 to below 1x 10^−10^ [M1 and M2]). Since activation in the hemisphere ipsilateral to the stimuli was weak or absent, only contralateral activation is shown: *Motion Left minus Stationary Left* is projected on the right STG, while *Motion Right minus Stationary Right* is projected on the left STG. Three different views of each STG are represented: a top, a lateral, and a bottom view allowing the visualization of the superior bank (Sup.), the convexity (Conv.), and the inferior bank (Inf.) of the STG, respectively. The transparent region within the black boundary corresponds to motion-responsive regions (defined by the contrasts *Motion Left minus Silence* and *Motion Right minus Silence*, P_FWE_ < 0.05) where the BOLD responses induced by motion and stationary stimuli were not significantly different. The numbers 1, 2, 3 and 4 indicate the locations where the plots illustrated in panel C are extracted from. (C) Hemodynamic response functions for motion and stationary stimuli in M1 (Experiment 2). The BOLD response (expressed by the response estimate coefficient, betas) was measured 2, 3, 4, and 5 s after the stimulus onset. The plots illustrate data from four representative individual voxels whose locations are indicated in panel B. ANOVA tests: *n* = 5,794, df = 4,905, F-values below 2.1, and all P_FWE_ = 1. Error bars correspond to standard errors. Data are available from the Open Science Framework (https://osf.io/ut5pa/).

In each subject (monkey 1, M1; monkey 2, M2), the contrasts *Motion Left minus Stationary Left* and *Motion Right minus Stationary Right* revealed widespread activation of the posterior part of the auditory cortex contralateral to the stimuli, on the superior temporal gyrus (STG) (Fig 2A and 2B). The activation included the three stages of the hierarchically organized auditory cortex, namely the core (in A1), the belt (in the middle lateral [ML] and CL areas, surrounding A1), and the parabelt (the caudal part of the parabelt, lateral to CL and ML, on the STG convexity), and extended into the inferior bank of the STG (also known as the superior bank of the superior temporal sulcus). Activation in the ipsilateral hemisphere was much more limited (M1) or absent altogether (M2). These results indicate that motion-induced activity in the contralateral posterior auditory cortex cannot simply be explained by a spatial laterality process or by encoding of the intrinsic spectrotemporal content of the moving stimuli since these processes were controlled for by the stationary stimuli.

While in Experiment 1 the BOLD response induced by each stimulus was measured 5 s after the stimulus onset (targeting the peak of the hemodynamic response function [28]), in a second experiment we measured the time course of the hemodynamic response induced by each stimulus, recording the BOLD response 2, 3, 4, and 5 s after the stimulus onset (Fig 2C). This was to eliminate the possibility that the greater activation triggered by motion stimuli in Experiment 1 was due to adaptation of the BOLD response to the stationary stimulus while the motion-induced response was still sustained at its maximal level. This experiment revealed no interaction between the two stimulus conditions and the four time points tested, except in a small cluster in the right inferior bank of the STG, where stationary sounds did not induce any significant activation. This confirms that the contrast between moving and stationary stimuli revealed in Experiment 1 was not due to a different time course of the BOLD response to the two types of stimuli.

### Experiment 3: Motion versus spectrotemporal control

**Fig 3:**
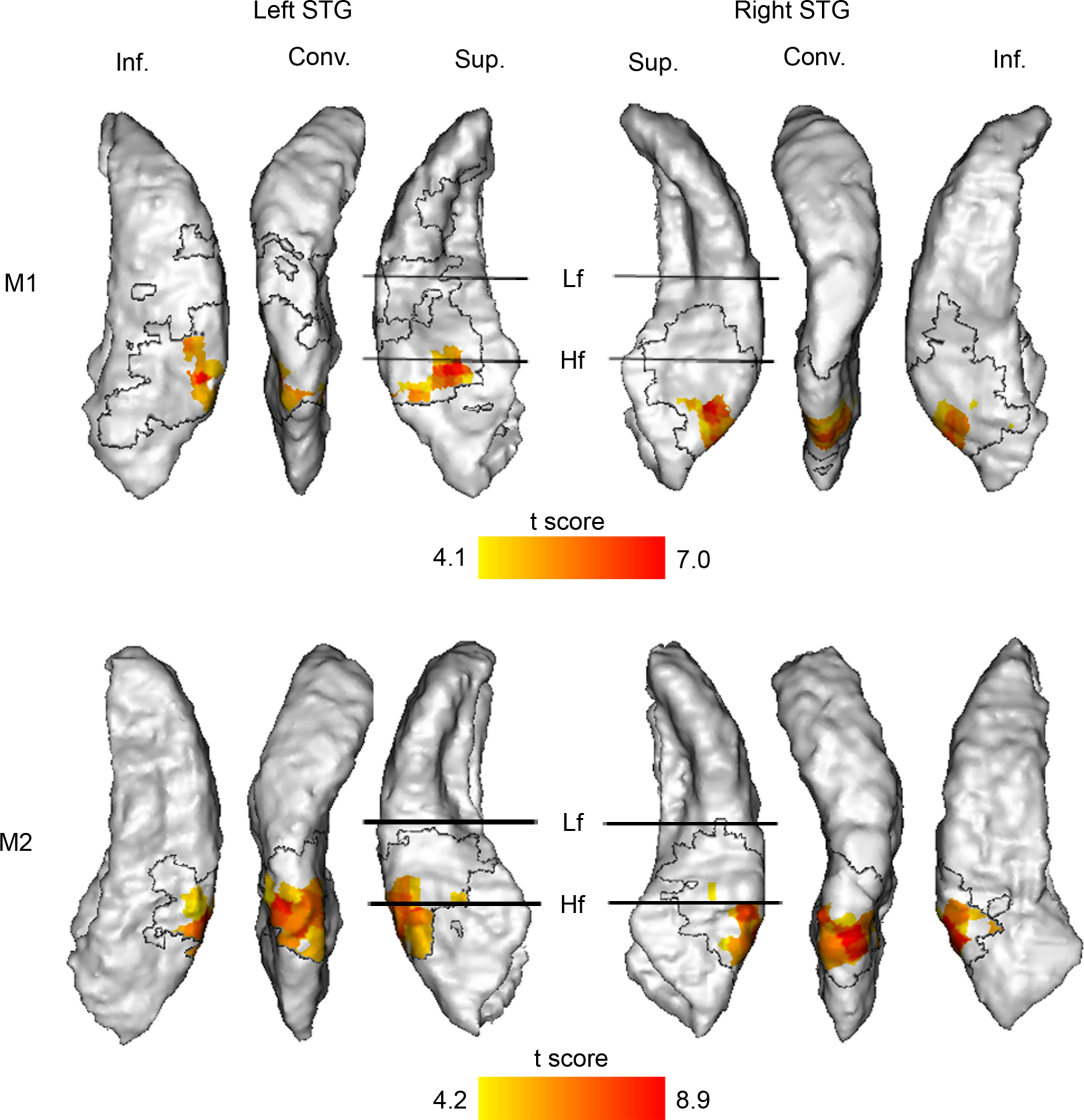
*Motion minus Spectrotemporal control* contrast (Experiment 3). Significant statistical parametric *t* maps are shown on a surface rendering of the superior temporal gyrus (STG) contralateral to the stimuli (no activation in the ipsilateral hemisphere): *Motion Left minus Spectrotemporal control* is projected on the right STG, while *Motion Right minus Spectrotemporal control* is projected on the left STG (*n* = 3,028 [monkey 1, M1] and 2,664 [monkey 2, M2]; degrees of freedom [df] = 2,722 [M1] and 2,382 [M2]; P_FWE_: from 0.05 to 4 x 10^−9^ [M1] and from 0.05 to below 1 x 10^−10^ [M2]). The transparent region within the black boundary corresponds to motion-responsive regions (defined by the contrasts *Motion Left minus Silence* and *Motion Right minus Silence*, P_FWE_ < 0.05) where the blood-oxygen-level-dependent (BOLD) responses induced by motion and stationary stimuli were not significantly different. Conv., convexity; Hf, high frequency; Inf., inferior bank; Lf, low frequency; Sup., superior bank. For more details, see Fig 2 legend. Data are available from the Open Science Framework (https://osf.io/ut5pa/).

The change of spatial location inherent to moving stimuli induces some temporal variations of the sound spectral envelope due to filtering of the sounds through the pinna. This spectrotemporal effect of motion could theoretically be the source of auditory motion selectivity as described so far. To control for this, we generated a new stimulus by averaging at each time point the signal coming from each channel and presenting the stimulus diotically (i.e., the same signal was sent to each ear). This stimulus had a spectrotemporal structure similar to the motion stimulus, allowing us to control for the combined effect of the intrinsic spectrotemporal content of the stimulus and the spectrotemporal effect of motion, but did not contain any static spatial cue (Fig 1A). In our third experiment, we compared the BOLD response induced by motion with the one induced by the spectrotemporal control stimulus. The contrasts *Motion Left minus Spectrotemporal control* and *Motion Right minus Spectrotemporal control* revealed activation in ML, CL, the caudal parabelt, and the inferior bank of the STG in the hemisphere contralateral to the stimuli (Fig 3) and no activation in the ipsilateral hemisphere. The results thus indicate that motion-induced activation in these contralateral regions cannot be explained by spectrotemporal processes.

### Experiment 4: Motion = spectrotemporal + static spatial processes?

**Fig 4:**
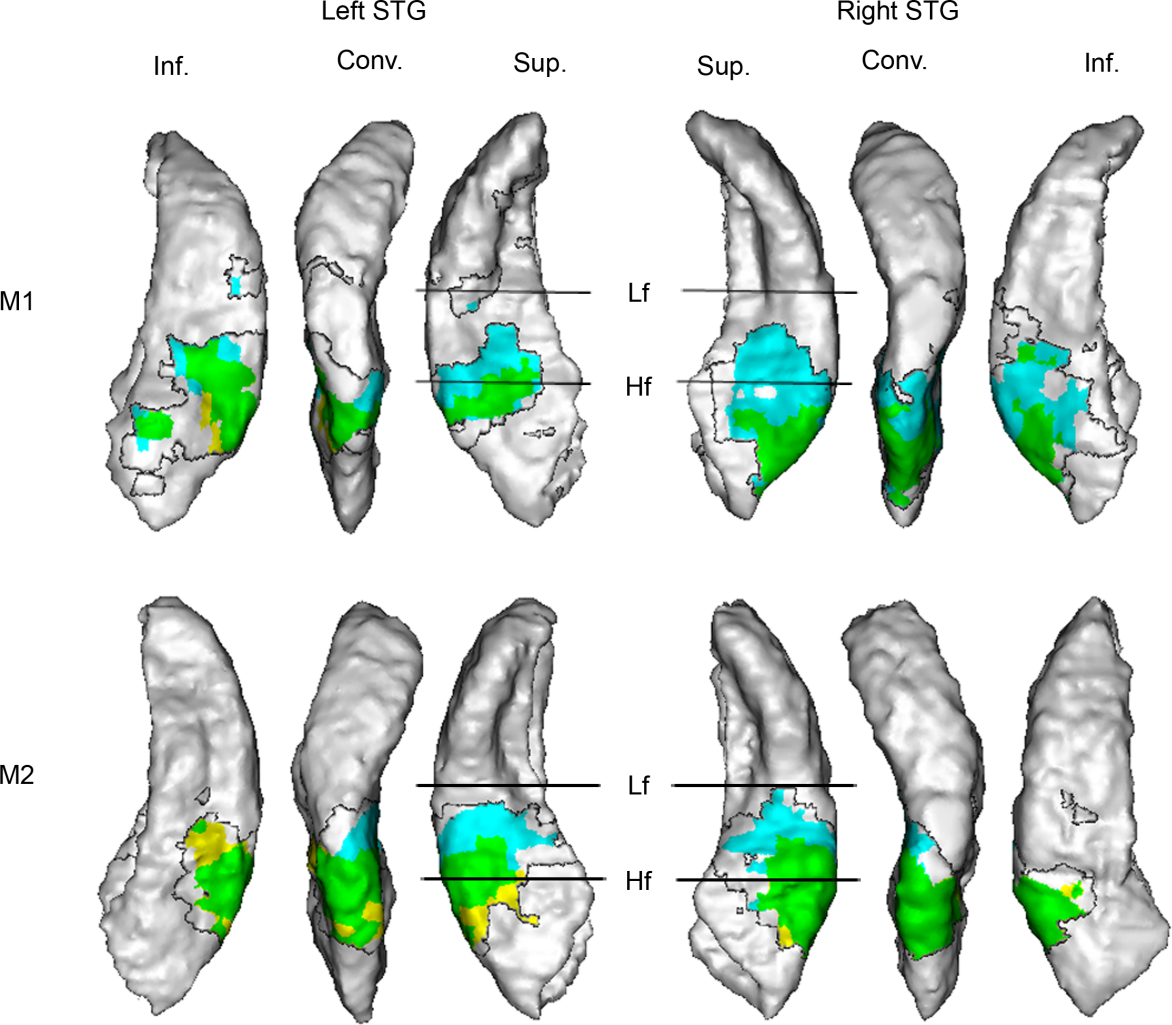
*Motion minus Stationary* contrasts (in cyan) and *Motion minus Spectrotemporal control contrasts* (in yellow) (Experiment 4). Voxels where each contrast is statistically significant (P_FWE_ < 0.05) are respectively colored in cyan and yellow (*t*-tests: *n* = 9,927 [monkey 1, M1] and 6,664 [monkey 2, M2]; degrees of freedom [df] = 8,902 [M1] and 5,922 [M2]). The green area corresponds to voxels where both contrasts are significant. Statistical results are shown on a surface rendering of the superior temporal gyrus (STG) contralateral to the stimuli: *Motion Left minus Stationary Left/Spectrotemporal control* is projected on the right STG, while *Motion Right minus Stationary Right/Spectrotemporal control* is projected on the left STG. The transparent region within the black boundary corresponds to motion-responsive regions (defined by the contrasts *Motion Left minus Silence* and *Motion Right minus Silence*, P_FWE_ < 0.05) where the blood-oxygen-level-dependent (BOLD) responses induced by motion and stationary stimuli were not significantly different. Conv., convexity; Hf, high frequency; Inf., inferior bank; Lf, low frequency; Sup., superior bank. For more details, see Fig 2 legend. Data are available from the Open Science Framework (https://osf.io/ut5pa/).

We then tested the hypothesis that a linear combination of spectrotemporal and stationary spatial processes could fully explain motion-related activity. To do so, we gathered in a single experiment the three different types of stimuli: motion, stationary, and spectrotemporal control stimuli. As a control, we first computed the same contrasts as in Experiments 1 and 3: the contrasts *Motion minus Stationary* and *Motion minus Spectrotemporal controls* revealed patterns of activation similar to those observed in Experiments 1 and 3 (Fig 4). Brain regions commonly activated by both contrasts were ML, CL, the caudal parabelt, and parts of the inferior bank of the STG (green region in Fig 4). This result indicates that in these regions, motion-related activation cannot be explained by static spatial processing (controlled in the contrast *Motion minus Stationary*), spectrotemporal effect of motion (controlled in the contrast *Motion minus Spectrotemporal control*), or the intrinsic spectrotemporal content of the stimulus (controlled in both contrasts) alone.

**Fig 5:**
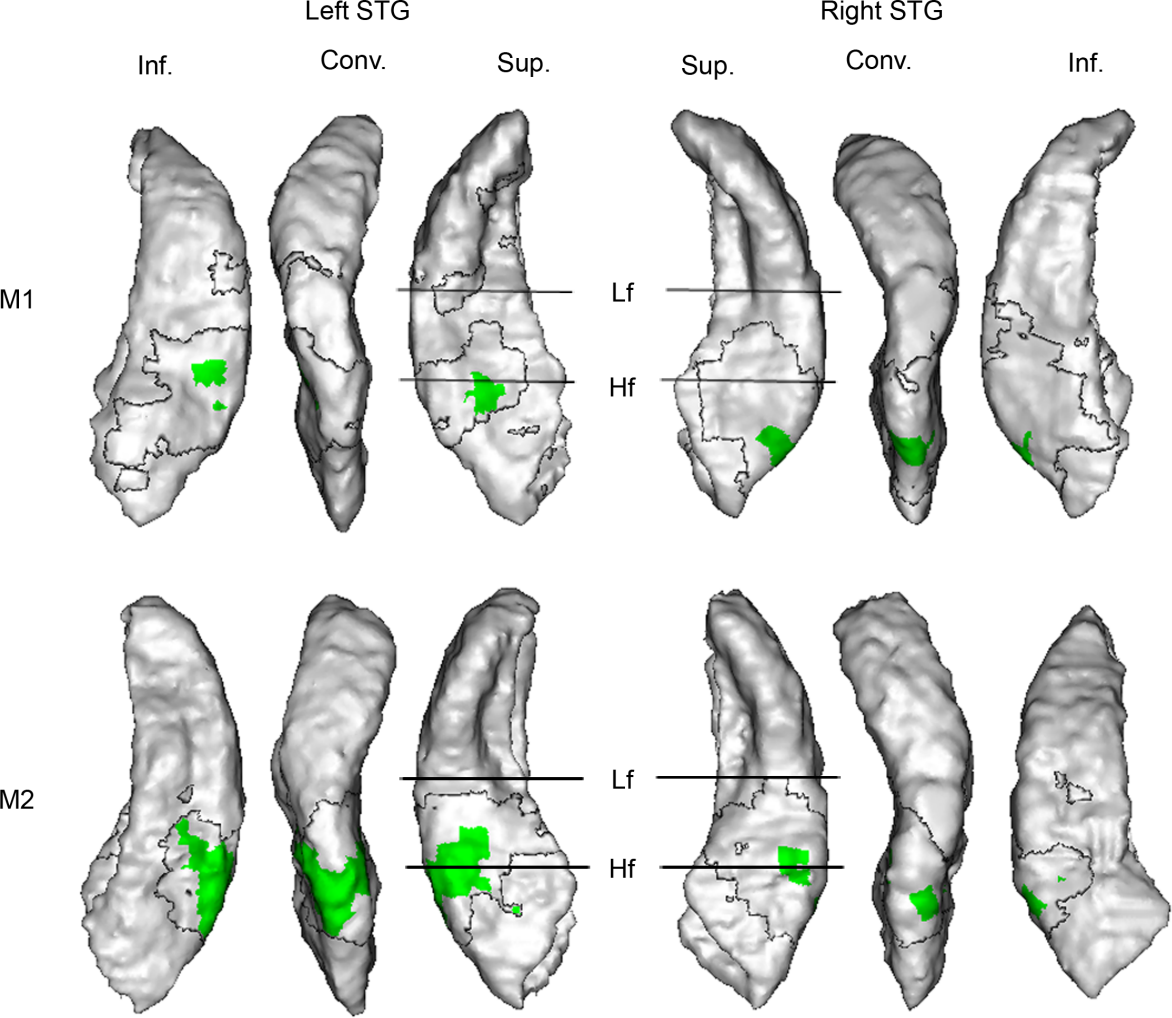
*Motion minus (Stationary central sound + Spatial laterality + Spectrotemporal effect of motion)* contrast (Experiment 4). Voxels where the contrast is statistically significant (P_FWE_ < 0.05) are colored in green (*t*-tests: *n* = 9,927 [monkey 1, M1] and 6,664 [monkey 2, M2]; degrees of freedom [df] = 8,902 [M1] and 5,922 [M2]). Statistical results are shown on a surface rendering of the superior temporal gyrus (STG) contralateral to the stimuli. The transparent region within the black boundary corresponds to motion-responsive regions (defined by the contrasts *Motion Left minus Silence* and *Motion Right minus Silence*, P_FWE_ < 0.05) where the blood-oxygen-level-dependent (BOLD) response induced my motion was not significantly different than the additive model. Conv., convexity; Hf, high frequency; Inf., inferior bank; Lf, low frequency; Sup., superior bank. For more details, see Fig 2 legend. Data are available from the Open Science Framework (https://osf.io/ut5pa/).

We then tested the hypothesis that the addition of these processes could explain motion-induced activation. More specifically, we tested the following model: Motion = Static central sound + Spatial laterality + Spectrotemporal effect of motion, where spatial laterality was defined with the contrast *Stationary sounds minus Stationary central sound* and the spectrotemporal effect of motion with the contrast *Spectrotemporal controls minus Stationary central sound* (S2 Fig). Motion-induced activation was not found to be significantly different from the sum of the three components in most parts of brain regions activated by motion stimuli, including A1 (Fig 5, transparent regions within the black boundary), indicating that the simple additive model provides a good estimation of the activation induced by motion stimuli in these regions. However, in parts of ML, CL, the caudal parabelt, and the inferior bank of the STG, the BOLD response induced by motion stimuli was significantly greater than the sum of the BOLD responses induced by stationary central sound, spatial laterality, and spectrotemporal effect of motion (Fig 5, green cluster).

The excess signal (i.e., the part not explained by the above components) that was present in these areas could arise if the three processes made different contributions (i.e., different weighting coefficients attached to each process), if the processes interacted, or if an additional, independent (and thus genuine motion-specific) process would trigger the response. To distinguish between these explanations, we performed a multiple linear regression analysis across those voxels where the BOLD response to auditory motion was not fully explained by the simple additive model (green cluster in Fig 5). This analysis revealed that the motion-induced BOLD signal was best estimated by a model including differential weighting factors (with a slightly smaller contribution of spectrotemporal effect of motion, compared to the two other components in both subjects; see Table 1), weak or no interactions between the components (R^2^ change between models with and without interactions: M1: 0.004, F = 0.13, *p* = 0.969; M2: 0.019, F = 4.09, *p* = 0.004; R^2^ for best model: M1: 0.82; M2: 0.86; *p*-values below 1 x 10^−6^ for each subject), and, importantly, a term that assumes the presence of genuine motion-selective responses to occur in these areas (the added constant term in the linear regression, Table 1, *p*-values below 1 x 10^−5^ for each subject). This additional component accounted for about 42% of the mean signal intensity induced by motion stimuli (M1: 42.5%; M2: 42.2%). A control analysis across voxels taken within the region where the full signal could be explained by the simple additive model (transparent patch in Fig 5) revealed similar regression coefficients but no significant constant term (Table 1). S3 Fig illustrates the goodness of fit of the best-adjusted model with experimental data in each subject.

**Table 1:** Multiple linear regression coefficients and associated statistics.

			Coefficient	SE	t/F	*p*
M1						
	*Inside green cluster*					
		Motion constant	9.0	1.51	6.0	3 x 10^−6^
		Stationary	1.2	0.14	8.4	<1 x 10^−6^
		Laterality	1.3	0.41	3.1	5 x 10^−3^
		ST	0.7	0.29	2.4	2 x 10^−2^
	*Outside green cluster*					
		Motion constant	-0.2	0.83	−0.2	0.84
		Stationary	1.1	0.07	15.3	<1 x 10^−6^
		Laterality	1.0	0.24	4.1	4 x10^-4^
		ST	0.6	0.07	7.6	<1x 10^−6^
M2						
	*Inside green cluster*					
		Motion constant	7.5	0.58	12.9	<1 x 10^−6^
		Stationary	1.4	0.07	19.2	<1 x 10^−6^
		Laterality	1.3	0.10	13.1	<1 x 10^−6^
		ST	0.8	0.07	11.5	<1 x 10^−6^
		Stationary x Laterality	0.03	0.02	1.6	0.12
		Stationary x ST	-0.05	0.01	−3.4	9 x 10^−4^
		Laterality x ST	-0.02	0.02	−1.4	0.18
		Stationary x Laterality x ST	0.001	0.004	0.4	0.72
	*Outside green cluster*					
		Motion constant	0.0	0.51	0.0	0.98
		Stationary	1.2	0.07	19.1	<1 x 10^−6^
		Laterality	1.0	0.10	9.9	<1 x 10^−6^
		ST	0.6	0.04	12.8	<1 x 10^−6^

“Inside green cluster” refers to the brain region depicted in green in Fig 5, where the contrasts *Motion minus Silence* and *Motion minus (Stationary central sound + Spatial laterality + Spectrotemporal effect of motion)* were significant. “Outside green cluster” refers to transparent voxels within the black boundary in Fig 5 and corresponds to voxels where the contrast *Motion minus Silence* was significant but where the contrast *Motion minus (Stationary central sound + Spatial laterality + Spectrotemporal effect of motion)* was not significant. Interaction coefficients and associated statistics are displayed only in the case where adding interaction terms significantly improved the model (statistically significant R^2^ change). Monkey 1, M1; monkey 2, M2; Motion constant, constant term of the linear regression; SE, standard error; ST, spectrotemporal effect of motion; stationary, stationary central sound process. See also S3 Fig.

### Experiment 5: Sensitivity of auditory motion areas to visual motion

**Fig 6:**
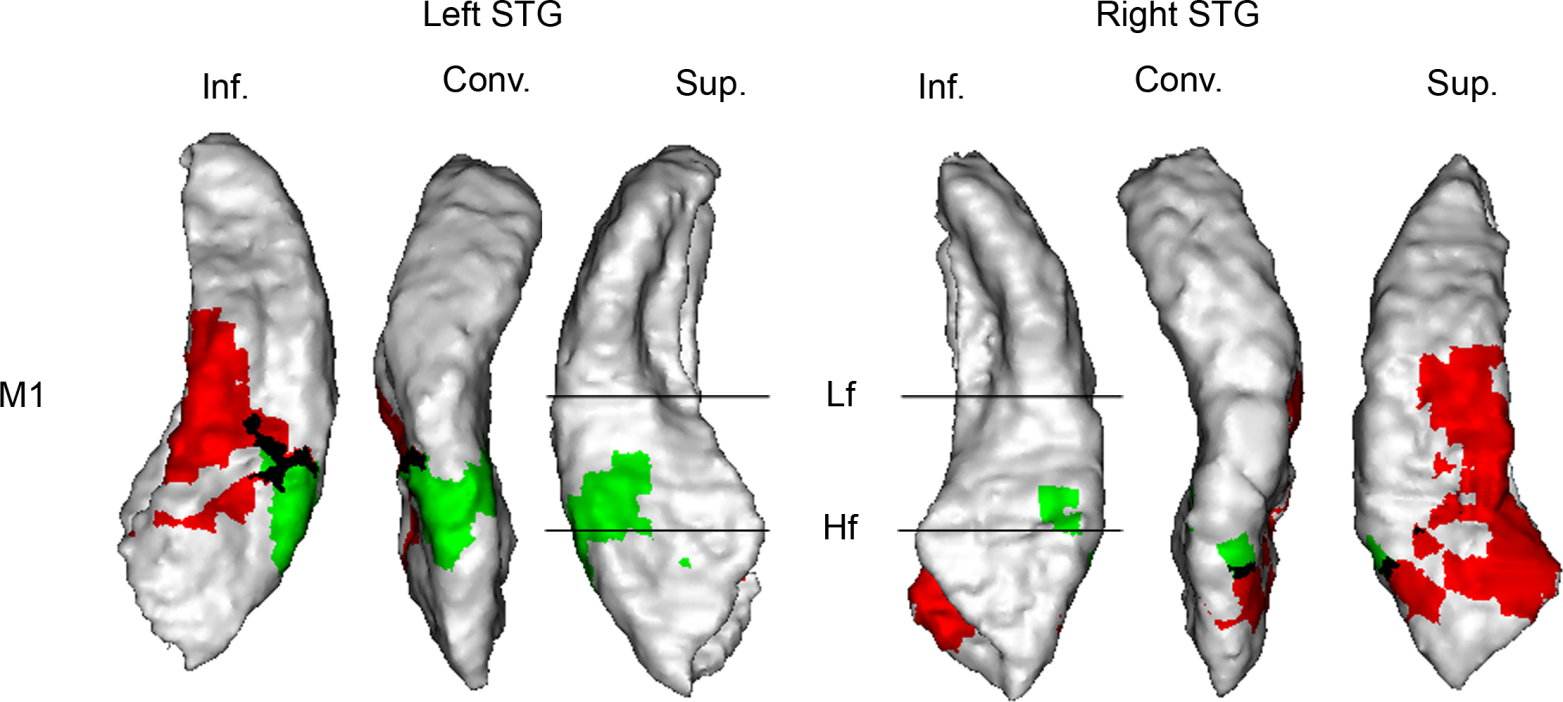
Auditory (in green) and visual (in red) motion-specific areas in monkey 2 (M2). Visual motion-specific areas were identified by the contrast *Moving gratings minus Stationary gratings* (Experiment 5, *t*-test: *n* = 1,241; degrees of freedom [df] = 1,067). Auditory motion data came from Experiment 4, and auditory motion-specific areas were identified by the contrast (*Motion minus (Stationary central sound + Spatial laterality + Spectrotemporal effect of motion)*) illustrated in Fig 5. The black area corresponds to voxels where both contrasts are significant. Significant statistical results (P_FWE_ < 0.05) are shown on a surface rendering of the superior temporal gyrus (STG) contralateral to the stimuli. Conv., convexity; Hf, high frequency; Inf., inferior bank; Lf, low frequency; Sup., superior bank. See Fig 2 legend for more details. Data are available from the Open Science Framework (https://osf.io/ut5pa/).

To further characterize these auditory motion-selective areas, we tested whether they were also selective for visual motion. Visual-motion areas were identified by contrasting the BOLD signal induced by slowly moving horizontally oriented gratings with that induced by stationary gratings. The auditory and visual motion regions were found to partially overlap on the inferior bank of the STG (Fig 6).

## Discussion

### Existence of motion-specific mechanisms

Movement selectivity has been classically investigated by comparing moving and stationary stimuli. In humans, the contrast *Motion minus Stationary* has consistently revealed activation of the planum temporale but not Heschl’s gyrus [20,21,29–32]. While human A1 has traditionally been considered to be located on the Heschl’s gyrus, recent tonotopy data suggest that A1 is rather found on the posterior half of the Heschl’s gyrus and slightly extends posteriorly into a small part of the planum temporale [33,34]. The planum temporale could thus encompass human homologues of macaque auditory areas CM, CL, and ML but also of the posterolateral part of A1. According to this model, our results fit well with human data since the contrast *Motion minus Stationary* induced the recruitment of the caudolateral parts of A1 and extended into CL and ML (Experiments 1 and 4).

The contrast *Motion minus Stationary* controls for the intrinsic spectrotemporal content of the motion stimuli and, when the stationary control sounds cover the whole spatial range spanned by the motion stimulus, its nondynamic spatial component as well. However, it does not control for the spectrotemporal effects of motion: filtering of sound through the pinna differs for each spatial position, inducing slow modulations of the sound spectral envelope. This dynamic nonspatial component aspect has only been controlled for in one previous study [21]. In this human fMRI study, spectrotemporal effects of motion were found to be processed in parts of the planum temporale overlapping with those involved in motion processing (revealed by the contrast *Motion minus Stationary*). Our study provides similar results in macaques (Fig 4).

Compared to the previous human study [21], our study in macaques went several steps further. First, we tested for the first time the possibility that the addition of the different components of auditory stimuli could explain the activation that they induced. Our results demonstrate that the additive combination of (1) the intrinsic spectrotemporal component (“Stationary central sound” processing), (2) the nondynamic spatial component (spatial laterality processing), and (3) the dynamic nonspatial component (spectrotemporal effect of motion) allows a full characterization of motion processing in large parts of the STG, including A1. However, in a circumscribed region overlapping parts of ML, CL, the caudal parabelt, and the inferior bank of the STG, the additive model did not explain a significant fraction of the signal induced by motion stimuli.

Second, we controlled for potential interactions between components. This was done by introducing in the linear models all possible interactions and testing whether the percentage of the variance explained by the model including the interactions was significantly higher than in the model without the interaction terms. This analysis revealed weak or no interactions between the components.

Third, we controlled for mechanisms that can influence the gain of the three different components (i.e., different forms of adaptation or amplification). It has been argued that greater activation induced by moving sounds compared to stationary sounds could represent adaptation of responses induced by stationary sounds [35–38]. By measuring the time course of the hemodynamic response induced by motion and stationary sounds, here we demonstrate that the differential activation cannot be explained by (slow) adaptation of the BOLD response triggered by stationary sounds (Experiment 2). Rapid adaptation of neuronal responses could also, in principle, explain greater activation induced by moving sounds. In the present study, the use of an amplitude-modulated noise as a stimulus should have limited such an effect. Moreover, adaptation or amplification mechanisms were controlled in the final linear regression analysis by the coefficients weighting the different components and their interactions. The regression analysis revealed that the magnitude of these mechanisms was moderate (coefficients close to 1) and similar between the region where part of the motion processing signal was left unexplained and the region where the signal could be fully explained by the simple additive model. Thus, adaptation or amplification fails to explain the unaccounted signal.

The linear regression analysis revealed that after controlling for the different processes not specific to motion, their potential interactions, and their potential adaptation or amplification, on average 42% of the signal variance remained unexplained. Since processes directly or indirectly linked to processing of several stationary sounds were controlled for in this analysis, we conclude that this remaining part of the signal comes from a motion-specific process.

The nature of the mechanisms underlying auditory motion perception has been debated for more than 30 y. The “snapshot hypothesis” postulates that motion is inferred from snapshots of object successive positions, without direct appreciation of motion. According to this hypothesis, auditory motion perception is based on the same mechanisms as those involved in the localization of static sound sources. The alternative hypothesis, usually referred as the “motion detector hypothesis” or “velocity detector hypothesis”, considers that motion perception is based on specific mechanisms. On the one hand, the fact that in humans, the minimum audible movement angle (MAMA; defined as the smallest movement angle allowing a subject to determine whether a sound is moving or not) differs from the minimum audible angle (MAA; defined as the smallest location difference between two static sources that subjects could discriminate) and was found to increase with speed has been interpreted as suggesting that motion detectors exist [3,4]. It has also been argued that if moving sounds are processed via a snapshot process, comparing the location of the starting and ending points should be sufficient to perceive movement, and information about intermediary locations should be redundant. However, the MAMA for moving sounds was found to be smaller than the MAA for tone bursts marking the starting and ending positions of the moving sound [5,39], and subjects could discriminate between accelerating and decelerating 90 ms-long stimuli starting and ending at identical spatial locations [6], indicating that the human brain extracts other information than the location of the starting and end points of a moving sound. On the other hand, movement detection and discrimination performances of human subjects have been explained by estimation of the distance traversed by the source rather than appreciation of the motion per se [1]. The MAMA and the MAA were also found to show similar dependency on sound frequency, spectral bandwidth, and source azimuth, suggesting that static spatial cue perception and dynamic spatial cue perception are dependent on the same underlying mechanisms [2,40]. Altogether, these data have failed to provide clear evidence about the mechanisms underlying motion perception. Our results indicate that the parts of the auditory cortex, including A1, analyze auditory sources in movement by processing their spatial location and the consequence of location change (spectrotemporal effect of motion), consistent with a “snapshot” strategy. However, we demonstrate that caudal belt and parabelt regions of auditory cortex extract the motion component of moving stimuli (motion-specific process) in addition to the non-motion specific components. The coexistence of the two mechanisms might explain why it has been so difficult to distinguish between the snapshot and the motion detectors hypotheses in the past. It is also relevant to psychophysical data illustrating facilitation of motion perception by static spatial information [7,8].

The BOLD response measured by fMRI only provides an indirect measure of neuronal activity. The choice of macaques as subjects of this study paves the way for a detailed investigation of the motion-specific mechanism at the cellular level. Our study indicates that motion-specific and snapshot processes coexist in the caudal belt and parabelt regions. Electrophysiological studies will be useful to determine whether the two types of processes are encoded by different populations of neurons or not.

This study investigated the mechanisms underlying auditory motion along the azimuth axis restricted to each hemispace. It is possible that the relative contribution of motion-specific and snapshot mechanisms depends on the nature of the movement (in elevation, in depth, or across hemispaces). Primates are particularly accurate at discriminating the spatial location of sounds coming from the regions near the midline [41], and based on the main opponent-channel hypothesis [42], the firing rate of auditory neurons contains more information about the precise location of a sound source when it is near the midline, by opposition to the peripheral space. It is thus possible that the contribution of motion-specific mechanism for sounds moving across the midline is less important than when sounds move within one hemispace. Looming sounds are particularly relevant from a behavioral point of view, often indicating a threat. In such sounds, information about the successive static position of the sound is limited to monaural cues and the distance or time to arrival is systematically underestimated [43]. Thus, one might expect the relative contribution of snapshot processes to be less important. These will be interesting hypotheses to test in the future.

### A further auditory stream?

Auditory information has been proposed to be processed along two main streams: a ventral stream connecting the rostral belt and parabelt areas to the ventral prefrontal cortex, involved in the identification of sounds, and a dorsal stream connecting the caudal belt and parabelt to the posterior parietal cortex and the dorsolateral prefrontal cortex, involved in spatial processing [18,44]. The exact number and the respective role of each stream are still a matter of debate. For instance, several authors have suggested that the auditory dorsal stream could be divided into distinct substreams [15,45–47]. Anatomical tract-tracing studies suggest at least two substreams originating from the caudal belt areas: a “dorsodorsal substream” involving CM, projecting to Tpt posterior to CM and thence to the parietal and prefrontal cortex; and a “dorsocaudal substream” connecting ML and CL to the caudal parabelt and the inferior bank of the STG, which itself projects to the parietal cortex [48].

Evidence for a role of the dorsal auditory pathway in spatial processing in nonhuman primates comes from electrophysiological data indicating that sensitivity to static spatial information is higher in CL compared to A1 and the rostral belt areas [15,18,19]. Our study revealed a similar refinement of static spatial processing between A1 and CL (see S4 Fig). Some single-unit electrophysiological studies also highlighted the role of CM in spatial processing [17,19]. However, spatial selectivity seems to be weaker compared to CL [19], and modelling of neural data suggests that the firing rates of CL neurons, but not of CM neurons, carry enough information to account for sound localization performance in azimuth [16]. Using fMRI, we did find strong and consistent BOLD activation induced by static and moving sounds in CL but not in CM. Since the BOLD response indirectly reflects the activity of very large populations of neurons, it is possible that at this scale, the spatial sensitivity of CM neurons cannot be detected. Together, these results suggest that spatial sensitivity differs to some extent between CL and CM and that the specialization for static auditory spatial processing mainly occurs along the dorsocaudal substream.

In addition to this static spatial processing, our data demonstrate a particular specialization for motion analysis within the dorsal-caudal substream, indicating that this stream carries out higher-level spatial computation rather than just representing fixed space. This result indicates the existence of parallel pathways for fixed and dynamic auditory spatial analysis within the dorsocaudal stream that likely feed into distinct downstream mechanisms as in the visual system. The exact number of substreams within the visual dorsal pathway and their respective roles are still debated [49–51]. Subdivision of the human visual dorsal pathway into at least two substreams has been proposed, with the dorsodorsal pathway involving the superior parietal lobule, while the ventral-dorsal pathway involves the visual motion areas in the temporal sulcus (including MT) and the inferior parietal region [52,53]. Auditory motion-specific areas described in the present study have been found to extend on the inferior bank of the STG (equivalent of the superior bank of the temporal sulcus), in the vicinity of visual motion areas, and our visual experiment revealed a small overlap between auditory and visual motion-selective areas in this region. These data suggest that the auditory and visual motion substreams share some neural substrates in the inferior bank of the STG, which might potentially support the numerous behavioral interactions that have been reported between auditory and visual motion perception [54,55]. It will be interesting to determine whether this region represents the point where the auditory and visual motion substreams merge by testing whether the same population of neurons respond to auditory and visual motion stimuli and where these neurons project.

Because the posterior auditory cortex in humans is involved not only in spatial analysis but also in speech and music perception, recent models of the dorsal auditory stream incorporate the idea that there may be a transformation of auditory information into a motor signal coding for the action necessary to produce the sound [47,56,57]. While this auditory-motor function could coexist with perceptual spatial processing in distinct substreams [47], it has also been argued that spatial processing could be interpreted as a preparation for eye movement or grasping [58,59]. In this case, the whole dorsal pathway could be characterized as a mechanism for auditory-motor integration, with different substreams supporting different auditory-motor processes. We suggest that the ability to compute motion allows a substream of the auditory dorsal pathway to predict the trajectory of sources in a way that helps visual tracking and grasping.

## Materials and methods

### Ethics statement

All procedures were approved by the Animal Welfare and Ethical Review Body at Newcastle University and by the United Kingdom Home Office (PPL 60/4095, 60/4037 and 70/7976). Experiments complied with the Animal Scientific Procedures Act (1986), the European Directive on the protection of animals used for scientific purposes (2010/63/EU), and the United States National Institutes of Health Guidelines for the Care and Use of Animals for Experimental Procedures and were performed with great care to ensure the well-being of the animals.

### Subjects

Two awake male rhesus monkeys (*Macaca mulatta*) M1 and M2, respectively 7 and 11 y old (weighing 7 and 17 kg), participated in the experiments. The monkeys were initially implanted with a head holder. All surgical procedures were performed under general anesthesia and sterile conditions. Details regarding surgical procedures, postoperative care, and the cleaning of the implant are published elsewhere [60]. The animals were first habituated to the scanner environment over the course of several days and then enrolled in the experiments.

### Auditory motion and control stimuli

Sound stimuli were created in MATLAB 7.1 (MathWorks, Natick, Massachusetts, US) with a sample rate of 44.1 kHz and 16-bit resolution. All stimuli for the auditory motion experiments (Experiments 1 to 4) were based on a random-phase noise carrier (1–20 kHz). The noise was amplitude modulated by a sinusoidal envelope of 80% depth at 80 Hz in order to produce an additional localization cue [61] and to prevent adaptation. Prior to the scanning experiments, the amplitude-modulated noise was delivered in free field in an echo-suppressed room from 17 different positions separated by 10°, along the azimuthal axis (from −80° to +80°) and recorded with a omnidirectional miniature electret microphone (Knowles Corporation, Itasca, Illinois, US) placed within each ear canal of the subject, resulting in the recording of the sound convolved by the head-related transfer function of each monkey. The microphone output was amplified and recorded digitally at a sampling rate of 44.1 kHz. In addition to interaural level and time differences, this whole procedure preserves spectral cues specific to each individual and has been shown in humans to induce stimuli to be perceived as localized in the external space when delivered through headphones [62]. While providing stimuli tailored to each subject, this approach is time consuming, and when applied to macaques, it is limited by the fact that the subject’s pinnae are mobile such that movements can distort spectral cues. By gently holding the subject’s pinnae from the back and keeping the stimulus recording sessions short (~1 h), we could prevent any movement. To accommodate this time limitation, we did not attempt to record sounds separated by less than the MAA (around 3° in macaques; see [17,26,63]). Instead, we took advantage of the fact that a motion percept can be induced by sequentially presenting sounds coming from spatial positions separated by more than the MAA as long as these spatial positions are not too far apart and as long as the duration of each sound is short enough (in other words, as long as the apparent speed is high enough). Motion stimuli moving three times back and forth between the central space (0° position) and the most lateral position (+ or −80° position) within each hemispace were created by concatenating 100 ms-long segments of recordings from adjacent locations (Fig 1); half of the stimuli started from the central position and the other half started from the most lateral position. Any abrupt change of power between segments was avoided by concatenating on-phase segments, starting and finishing when the power of the amplitude-modulated signal was minimum. Since these spatial locations were separated by 10°, it resulted in stimuli virtually moving at a speed of 100°/s. A similar approach was used to create stationary stimuli: 100 ms-long recorded segments coming from the same location were concatenated to form long examplars of stationary sounds. Five different stationary stimuli were created, corresponding to spatial positions −80°, −40°, 0°, +40°, and +80° (Fig 1). Finally, spectrotemporal controls of each motion stimulus were created by averaging at each time point the signal coming from each channel and presenting the stimulus diotically (Fig 1). The resulting stimuli were sounds with intensity and spectral content varying with time like the motion stimuli but with no spatial information (stimuli sounding as coming from inside the head, without spatial laterality). All auditory motion and control stimuli were 4.9 s long.

### Validation of stimulus motion

To validate the percept induced by the virtual motion stimuli in our monkey subjects, we characterized our stimuli psychophysically in humans. This approach takes advantage of the similarity of spatial perception between humans and monkeys and of the fact that the MAA is actually smaller in humans [17,26,27,63], allowing us to establish a more exacting test in which differences between concatenated and moving stimuli are more likely to be detected. Briefly, we built an apparatus capable of delivering static or moving sound stimuli in free field in our soundproof chamber. This used an electric motor with adjustable speed (controlled by a potentiometer) with an attached rotor arm to which a small speaker was attached, to achieve sound-source rotatory movement in the azimuthal plane through the subject’s ear canal. We have replicated in three human participants (two males and one female with no hearing disorder, age range: 20–35 y, having given their informed consent) the intra-auricular recording approach used in macaques, using exactly the same sounds and the same recording equipment. For each human participant, we recorded from the ear canal when static sounds were delivered from azimuthal positions recorded in 10° intervals from zero (midline, front). Additionally, we recorded motion stimuli from the ear canal when a speaker moved around the head with an angular motion of 100°/s or 50°/s, clockwise and anticlockwise. The two types of recordings were based on the same amplitude-modulated noise stimulus used in the macaque work. The recording session lasted between 2 and 3 h, requiring the human participant to remain still during this period. Static recordings from adjacent positions were then concatenated to create stimuli virtually moving at speeds of 100°/s or 50°/s, the duration of each recorded segment being 100 and 200 ms, respectively (concatenated stimuli). This procedure was used to create four concatenated stimuli per participant, while recordings of real moving sounds were used to create moving stimuli matching the travelled path and the direction of the concatenated stimuli: two stimuli of each type moved clockwise (one moving from +90° to +180° and the other one moving from −90° to 0°), and two stimuli moved anticlockwise (one moving from −90° to +180° and the other one moving from 90° to 0°). We tested each participant’s perception of these stimuli using criterion-free psychophysics. We used an AXB psychophysical paradigm, where X was always a moving stimulus and A and B were either a moving stimulus or a concatenated stimulus (whether A or B was the moving stimulus was randomized across trials) and each stimulus moved at 100°/s along the same path. Participants were asked to identify which of stimuli A or B was different from X. The results confirmed that no participant was able to distinguish concatenated stimuli from motion stimuli at 100°/s (performance was at chance level in each participant: Chi-square tests, *n* = 240, degrees of freedom [df] = 1, X^2^/*p* = 0.6/0.44 [participant 1], 0.42/0.52 [participant 2], 0.6/0.44 [participant 3]; S5 Fig). Because spatial acuity in azimuth is better in humans than macaques [17,26,27,63], this result supports our claim that concatenated stimuli used in the scanner were perceived by macaques as smoothly moving. In a control experiment, we replicated the AXB psychophysical paradigm using the concatenated and motion stimuli moving at a speed of 50°/s. This second experiment confirmed that the percept is speed dependent, as two participants were then able to discriminate concatenated stimuli from motion stimuli (Chi-square tests, *n* = 240, df = 1, X^2^/*p* = 138/<1 x 10^−19^ [participant 1], 86.4/<1 x 10^−19^ [participant 3]), while the third participant was still at chance level (Chi-square test, *n* = 240, df = 1, X^2^/*p* = 0.42/0.52, S5 Fig).

### Other stimuli

Stimuli for the tonotopy experiment were based on a random-phase noise carrier with three different passbands, 0.5–1 kHz, 2–4 kHz, and 8–16 kHz, resulting in three different stimuli that encompassed different spectral ranges. The carriers were amplitude modulated with a sinusoidal envelope of 90% depth at 10 Hz to achieve robust responses.

Stimuli for the visual motion localizer experiment (Experiment 5) were horizontally oriented gratings (spatial frequency: 0.5 cycles/°) of 6° diameter, displayed at 7° to the right or to the left of the vertical meridian. Half of the stimuli were moving at a frequency of 8 Hz, while the remaining stimuli were stationary.

### Auditory stimuli delivery

Auditory stimuli were delivered in the scanner at an RMS sound pressure level of 74 dB using custom adapted electrostatic headphones based on a Nordic NeuroLab system (Nordic NeuroLab, Bergen, Norway). These headphones feature a flat frequency transfer function up to 16 kHz and are free from harmonic distortion at the applied sound pressure level. We recorded the spontaneous eye movements of one monkey when exposed to the stationary lateralized stimuli (−80°, −40°, +40°, and +80°) through the headphones. Perception of the stimuli induced systematic eye movements in the direction of the sound (S6 Fig), indicating that the spatial information of the stimuli was preserved through the headphones and that the monkey could perceive it.

### Experimental design

Subjects were scanned in a sitting position, head-fixed, while engaged in a visual fixation task (fixation window: 2°). Eye position was monitored at 60 Hz with a camera-based system (SensoriMotoric Instruments, Teltow, Germany), and correct fixation was rewarded by drops of fruit juice.

To avoid any contamination of the stimulus-induced BOLD responses by the response evoked by the acoustic noise of the scanner, a sparse-sampling paradigm was used for all auditory experiments. Images were acquired every 10 s (acquisition time: 1.6 s), stimuli being presented during the 8.4 s silent gap. Based on a previous time course characterization of the BOLD response in the auditory system of macaques [28], the plateau phase of the BOLD response was targeted in experiments 1, 3, and 4 by starting acquisition of the images 5 s after the stimulus onset. In Experiment 2, image acquisition started 2, 3, 4, and 5 s after the stimulus onset. To obtain baseline data, stimuli were omitted in 25% of the trials prior to image acquisition. The visual localizer experiment was acquired with a continuous paradigm. Stimuli were delivered in a pseudorandomized way, ensuring that each stimulus was presented the same number of times within each daily session. Because we aimed to only analyze trials in which the monkey was fixating, we interrupted the session when the monkey stopped fixation for more than 5 min. The number of trials per stimulus therefore varied from one session to the next, according to the monkey’s willingness to participate in the visual fixation task. Between 28 and 50 images per stimulus were acquired in each daily session. For the tonotopy experiment, 3 sessions were acquired in each subject. For the first, third, and fourth auditory motion experiments, 5, 6, and 17 sessions were acquired in M1, and 7, 6, and 14 sessions were acquired in M2. For the hemodynamic response function experiment (Experiment 2), 11 sessions were acquired in M1. For the visual localizer experiment (Experiment 5), 7 sessions were acquired in M2.

### Imaging data acquisition

Data were recorded in a 4.7 T actively shielded vertical MRI scanner (Bruker Biospec 47/60 VAS) equipped with an actively shielded gradient system (Bruker GA-38S) of 38 cm innerbore diameter (Bruker BioSpin, Ettlingen, Germany). A transmit/receive volume RF coil with an active decoupler (Bruker) was used to acquire functional and nonisotropic structural data. The volume coil in the transmit-only mode and an 8-channel receiving surface phased-array coil (H. Kolster, Windmiller Kolster Scientific, Fresno, California, US) were used to acquire isotropic structural data in order to generate three-dimensional surfaces.

Nonisotropic structural T1-weighted images (resolution: 0.5 mm x 0.5 mm x 2 mm) were acquired at the end of each session using a modified driven equilibrium Fourier transform (MDEFT) sequence with the same slice geometry as the functional scans to simplify coregistration. The imaging parameters were as follows: FOV: 12.8 cm x 9.6 cm; FA: 30°, TI: 800 ms; TE: 6 ms, TR: 2 s. Isotropic structural T1- and T2-weighted images (FOV: 10 cm x 10 cm; resolution: 0.6 mm x 0.6 mm x 0.6 mm) were acquired during a separate session using a magnetization-prepared rapid gradient-echo (MP-RAGE) sequence (FA: 27°, TI: 800 ms, TE: 7 ms, TR: 2.1 s), and a rapid acquisition with relaxation enhancement (RARE) sequence (TE: 14 ms, RARE factor: 8, TR: 5.5 s), respectively. No parallel acceleration was used.

Functional data covering the whole STG were acquired with a single-shot gradient-echo echo-planar imaging (EPI) sequence optimized for each monkey. Typical parameters were as follows: FOV: 12.8 cm x 9.6 cm; FA: 90°, TE: 21 ms, TA: 1.6 s, axial orientation, slice thickness: 2 mm, interleaved slice acquisition. The inplane resolution was 1 mm x 1 mm for M1 and 1 mm x 1.5 mm for M2.

### Data analysis

Functional MRI data were analyzed with SPM8 (http://www.fil.ion.ucl.ac.uk/spm/). Data acquired from each animal were processed separately in their native space. Images from each session were first realigned to the mean EPI image. No attempt was made to coregister EPI and structural scans. Instead, a pair consisting of a mean EPI image and a nonisotropic structural scan acquired during the same session was chosen as a reference, based on the quality of their alignment to each other. All functional images were coregistered to this reference EPI image, and all the nonisotropic structural scans were coregistered to the corresponding structural scan. The isotropic structural scans were coregistered to the mean of all nonisotropic structural scans. Functional data were smoothed with a kernel of 2 mm fullwidth at half maximum, high-pass filtered with a cut-off of 300 s to account for slow signal drifts, and adjusted for global signal fluctuations (global scaling).

In a general linear model analysis for the combined sessions of each experiment, the voxel-wise response estimate coefficients (beta-values) and *t*-values (one sided *t*-test) for the different contrasts of interest were calculated (head movement parameters were regressed out). Associated *p*-values were corrected for multiple comparisons using the FWE correction on the bilateral STG (Experiments 1, 2, and 3) and for the STG contralateral to the stimuli (Experiments 4 and 5). For auditory and visual motion experiments, data acquired while subjects did not fixate were discarded. For tonotopy experiments, all data were used.

In Experiment 4, a multiple linear regression analysis was performed across voxels in each subject using SPSS (IBM SPSS Statistics 21.0). The analysis was first performed across the set of voxels where the contrasts *Motion Left minus (Left Stationary central sound + Left Spatial laterality + Left Spectrotemporal effect of motion)* and *Motion Right minus (Right Stationary central sound + Right Spatial laterality + Right Spectrotemporal effect of motion)* performed at the voxel level were significant (*t*-values > 4.1, corrected *p* < 0.05). In order to determine the source of the signal that was not explained by the linear addition of components, we tested the following model across those voxels:

Motion = b1 x (Stationary central sound) + b2 x (Spatial laterality) + b3 x (Spectrotemporal effect of motion) + b4 x (Stationary central sound x Spatial laterality) + b5 x (Stationary central sound x Spectrotemporal effect of motion) + b6 x (Stationary central sound x Spatial laterality x Spectrotemporal effect of motion) + Motion constant term.

First- and second-order interactions between the three explanatory factors were incorporated only if this more complex model significantly increased the percentage of variance explained by the model. As a control, we performed the same analysis across a subset of voxels taken from the region where the voxel-based contrasts did not induce any significant difference (*t*-values < 4.1, corrected *p* > 0.05). To match the statistical power of both analyses, the size of this second voxel set was matched to the first one by selecting voxels with the smallest *t*-values. Since the probability of false-negative results decreases with the t value, this procedure reduced the risk of selecting false-negative voxels. Performing these analyses on smoothed and unsmoothed data provided similar results. Only results based on unsmoothed data are described in the Results section of the manuscript.

### Result displayed on 3D surfaces

Isotropic structural images were used to generate the rendered surfaces. The ratio between T1-weighted and T2-weighted images was computed, and the resulting image was used to manually segment the gray matter of the STG. The binary image was used to generate a tri-dimensional triangulated mesh using BrainVisa suite (http://brainvisa.info). The functional results (contrast maps and *t* maps) were then projected with BrainVisa onto the rendered surface.

### Tonotopy maps and localization of auditory areas

Tonotopy maps (S1 Fig) were calculated by subtracting the response estimate coefficient (beta-values) of the low-frequency condition (0.5–1 kHz) from the high-frequency condition (8–16 kHz). The contrast *High frequency minus Low frequency* was inclusively masked by the contrast *High frequency minus Silence*, while the contrast *Low frequency minus High frequency* was masked by the contrast *Low frequency minus Silence* (*p* < 0.05, uncorrected for multiple comparisons for both masks). The low- and high-frequency reversals of tonotopic gradients were identified on the surfaces and used to define the position of the core and belt areas on the superior bank of the STG: the low-frequency gradient reversal defined the rostral border of A1 and ML, and the high-frequency reversal defined the caudal border of A1 and ML and the rostral border of CL (see Fig 2A). The caudal parabelt was defined as the region lateral to ML and CL on the STG convexity.

## Supporting information

S1 Fig Tonotopy maps. Contrast maps, representing the degree of preference for high (cyan-blue colors) and low frequencies (red–yellow colors), are shown on a surface rendering of the superior bank of the STG.
(TIF)

S2 Fig Spectro-temporal effect of motion (in cyan) and spatial laterality (in yellow) (Experiment 4). Voxels where each contrast is statistically significant (P_FWE_ < 0.05) are respectively colored in cyan and yellow (t-tests: N = 9927 (M1) and 6664 (M2); df = 8902 (M1) and 5922 (M2)). The green area corresponds to voxels where both contrasts are significant 9. Statistical results are shown on a surface rendering of the superior temporal gyrus (STG) contralateral to the stimuli. Spectro-temporal effect of motion was assessed with the contrast *Spectro-temporal control minus Stationary central sound* and spatial laterality with the contrast *Stationary Left/Right minus Stationary central sound*. For more details, see Fig 2 legend.
(TIF)

S3 Fig Multiple linear regression plots inside and outside the motion-specific brain region (green cluster in Fig 5). The plots represent the relationship between the data estimated by the best adjusted model (Motion = b1 x (Stationary central sound) + b2 x (Spectro-temporal processes) + b3 x (Spatial laterality) + interactions + constant term) and the experimental data in each subject (M1 and M2). Data from both hemispheres have been merged for each subject. Inside the motion specific region. Number of voxels (n) = 30, F(3,26) = 40.1, p < 0.001 (M1), n = 130, F(3, 126) = 221.9, P < 0.001 (M2); outside the motion-specific region: n = 30, F(3,26) = 154, p < 0.001 (M1), n = 135, F(3.131) = 194.2, p < 0.001 (M2). For more details, see Table 1 legend.
(TIF)

S4 Fig Encoding of static spatial location. Significant statistical results (P_FWE_ < 0.05) are shown on a surface rendering of the superior temporal gyrus (STG) contralateral to the stimuli (N = 2466 (M1) and 3429 (M2); df = 2210 (M1) and 3071 (M2). Contra-hemisphere preference (in blue) was assessed by the contrast *Stationary Left minus Stationary Right*, projected on the right STG, and *Stationary Right minus Stationary Left*, projected on the left STG. These contrasts did not reveal any significant ipsilateral preference. The contrasts *Stationary central sound minus Stationary Right* and *Stationary central sound minus Stationary Left* did not reveal any preference for central sounds. Laterality preference (in red) was assessed by the contrasts *Stationary Left minus Stationary central sounds* and *Stationary Right minus Stationary central sound*. The black area corresponds to voxels where both contrasts were significant. This maps illustrate the refinement of static spatial processing between A1 and the dorso-caudal regions of the auditory cortex: while the broad location of static stimuli (left versus right hemispace) was encoded in large parts of the auditory cortex, including A1, the more precise location of the sounds (lateral versus central positions) was only processed in the most dorso-caudal regions (CL, caudal parabelt, inferior bank of the STG).
(TIF)

S5 Fig Human psychophysical data. Stimuli were moving stimuli and concatenated-static stimuli moving along one of four paths: from +90 to +180°, from -90 to 0°, from -90 to +180° and from 90 to 0°. Plotted data represents the percentage of correct responses over 240 trials that three human subjects made in the AXB psychophysical experiments. The dashed line represents chance level (50% of correct answer). *: significantly different from chance level using a chi-square test (fore detailed statistics, see text).
(TIF)

S6 Fig Monkey eye movement recordings. Eye movements when the subject was asked to not move his eyes (Fix, fixation spot on) and when subject heard the auditory stimulus while free to move his eyes (stimulus presentation, fixation spot off). Auditory stimuli were left (-80 or -40°) stationary stimuli (left) or right (40 or 80°) stationary stimuli (pink).
(TIF)

## Abbreviations

BOLD: blood-oxygen-level dependent
CL: caudolateral area
CM: caudomedial area
df: degrees of freedom
fMRI: functional magnetic resonance imaging
FWE: family-wise error
Hf: high frequency
Lf: low frequency
M1: monkey 1
M2: monkey 2
MAA: minimum audible angle
MAMA: minimum audible movement angle
ML: middle lateral area
STG: superior temporal gyrus
