# Supplementary figures and images for: Auditory motion-specific mechanisms in the primate brain
Source: PLoS Biol. 2017 May 4;15(5):e2001379. doi: 10.1371/journal.pbio.2001379 (PMC5417421; doi:10.1371/journal.pbio.2001379)

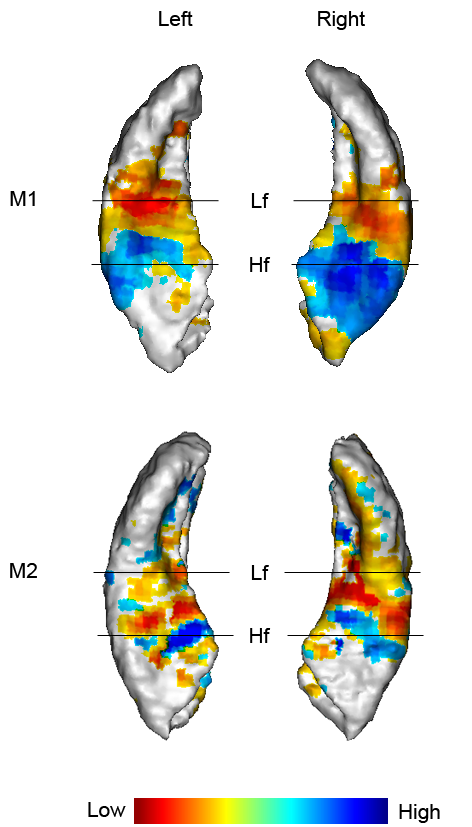

Supplement: S1 Fig — Contrast maps, representing the degree of preference for high (cyan-blue colors) and low frequencies (red–yellow colors), are shown on a surface rendering of the superior bank of the STG. (TIF) [file pbio.2001379.s001.tif]

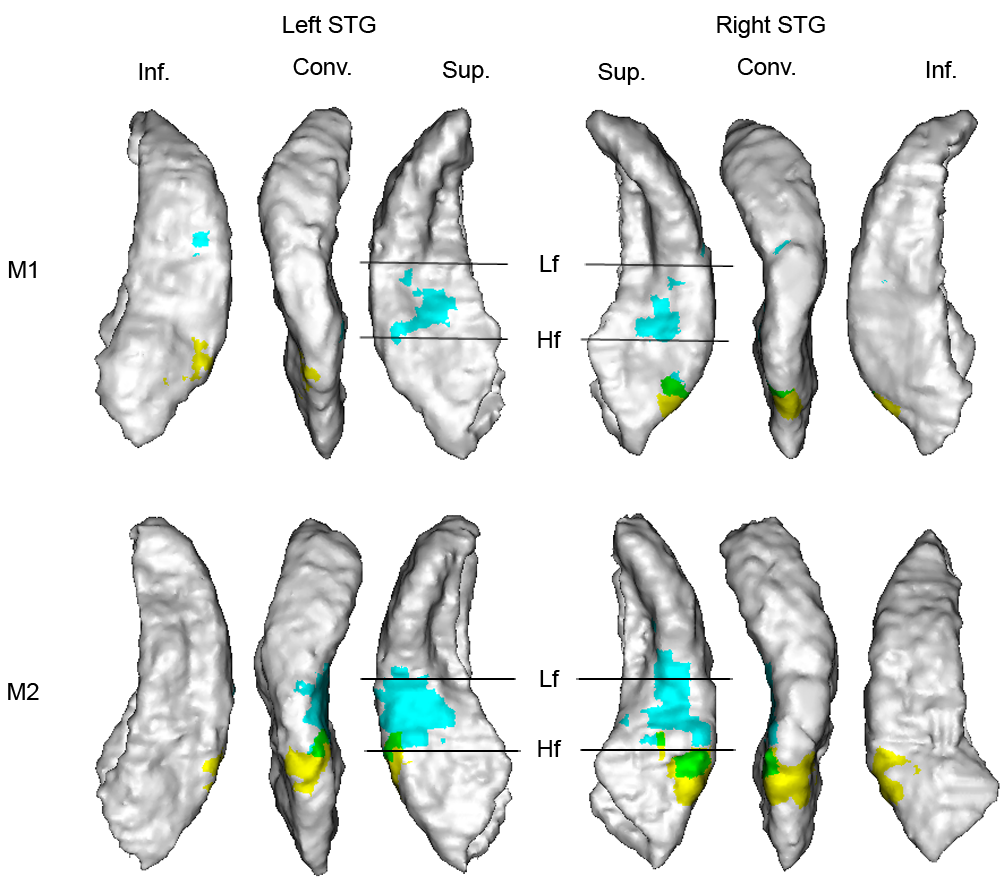

Supplement: S2 Fig — Voxels where each contrast is statistically significant (PFWE < 0.05) are respectively colored in cyan and yellow (t-tests: N = 9927 (M1) and 6664 (M2); df = 8902 (M1) and 5922 (M2)). The green area corresponds to voxels where both contrasts are significant 9. Statistical results are shown on a surface rendering of the superior temporal gyrus (STG) contralateral to the stimuli. Spectro-temporal effect of motion was assessed with the contrast Spectro-temporal control minus Stationary central sound and spatial laterality with the contrast Stationary Left/Right minus Stationary central sound. For more details, see Fig 2 legend. (TIF) [file pbio.2001379.s002.tif]

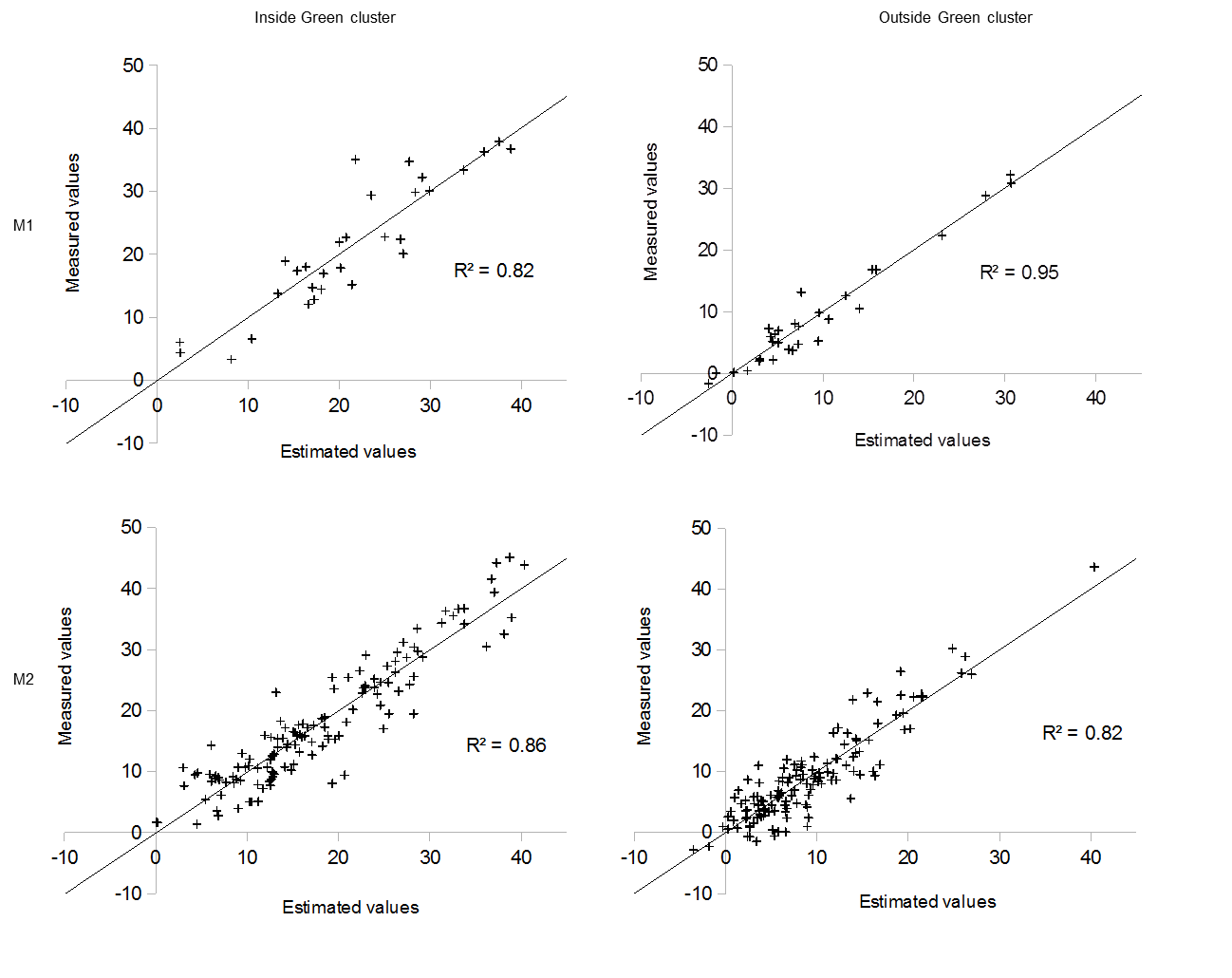

Supplement: S3 Fig — The plots represent the relationship between the data estimated by the best adjusted model (Motion = b1 x (Stationary central sound) + b2 x (Spectro-temporal processes) + b3 x (Spatial laterality) + interactions + constant term) and the experimental data in each subject (M1 and M2). Data from both hemispheres have been merged for each subject. Inside the motion specific region. Number of voxels (n) = 30, F(3,26) = 40.1, p < 0.001 (M1), n = 130, F(3, 126) = 221.9, P < 0.001 (M2); outside the motion-specific region: n = 30, F(3,26) = 154, p < 0.001 (M1), n = 135, F(3.131) = 194.2, p < 0.001 (M2). For more details, see Table 1 legend. (TIF) [file pbio.2001379.s003.tif]

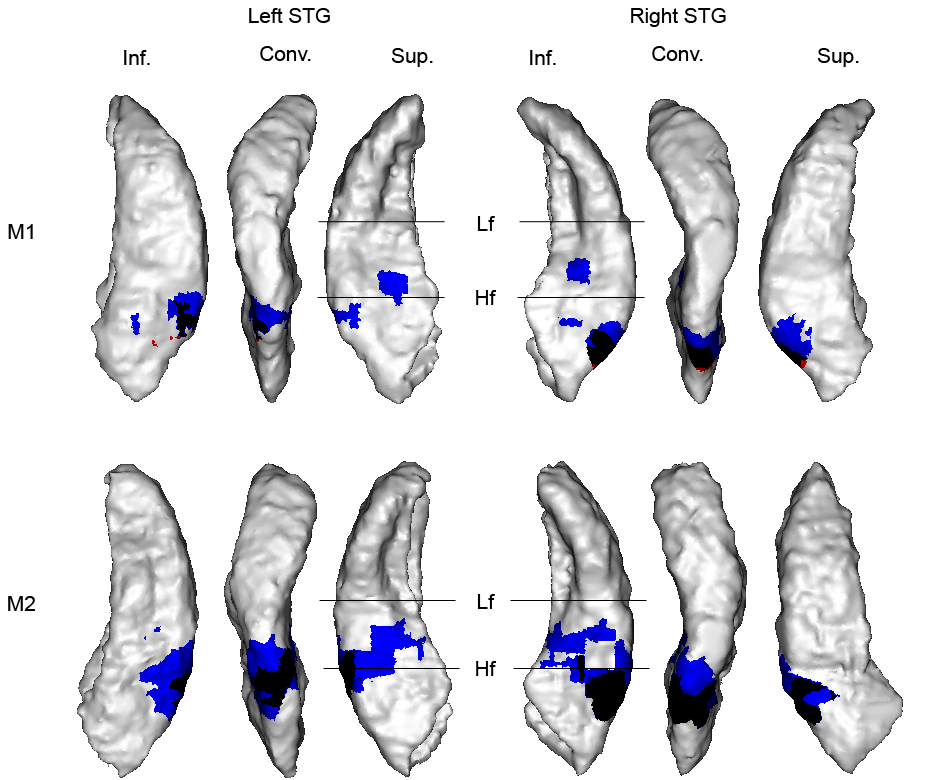

Supplement: S4 Fig — Significant statistical results (PFWE < 0.05) are shown on a surface rendering of the superior temporal gyrus (STG) contralateral to the stimuli (N = 2466 (M1) and 3429 (M2); df = 2210 (M1) and 3071 (M2). Contra-hemisphere preference (in blue) was assessed by the contrast Stationary Left minus Stationary Right, projected on the right STG, and Stationary Right minus Stationary Left, projected on the left STG. These contrasts did not reveal any significant ipsilateral preference. The contrasts Stationary central sound minus Stationary Right and Stationary central sound minus Stationary Left did not reveal any preference for central sounds. Laterality preference (in red) was assessed by the contrasts Stationary Left minus Stationary central sounds and Stationary Right minus Stationary central sound. The black area corresponds to voxels where both contrasts were significant. This maps illustrate the refinement of static spatial processing between A1 and the dorso-caudal regions of the auditory cortex: while the broad location of static stimuli (left versus right hemispace) was encoded in large parts of the auditory cortex, including A1, the more precise location of the sounds (lateral versus central positions) was only processed in the most dorso-caudal regions (CL, caudal parabelt, inferior bank of the STG). (TIF) [file pbio.2001379.s004.tif]

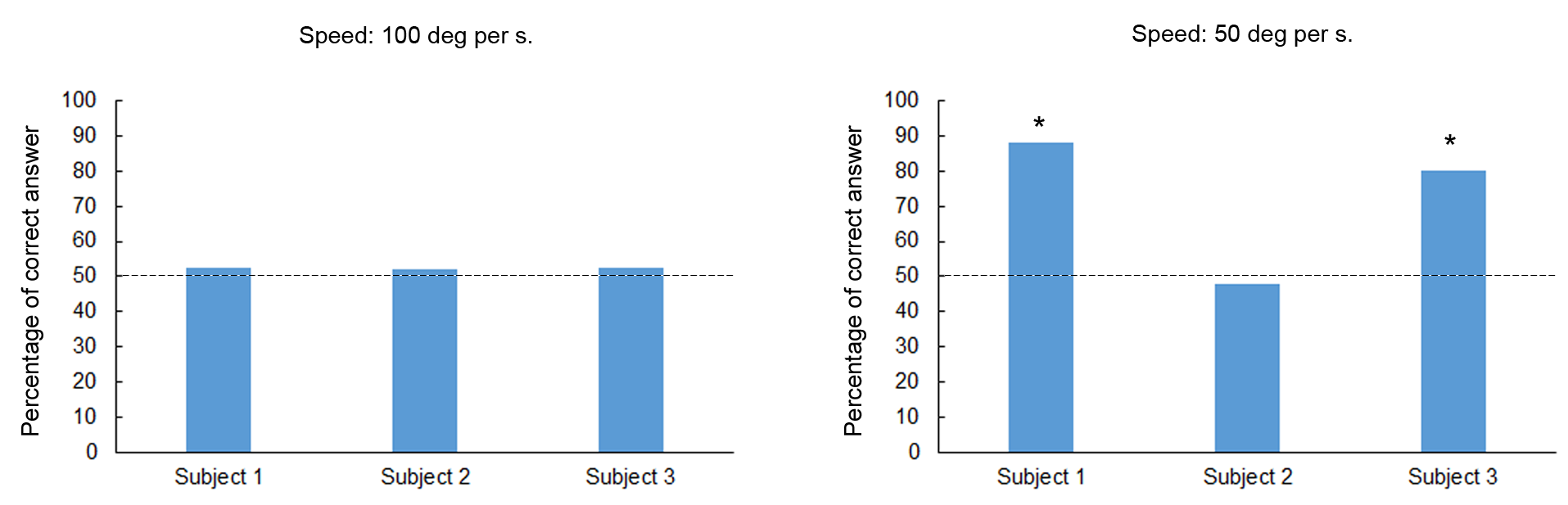

Supplement: S5 Fig — Stimuli were moving stimuli and concatenated-static stimuli moving along one of four paths: from +90 to +180°, from -90 to 0°, from -90 to +180° and from 90 to 0°. Plotted data represents the percentage of correct responses over 240 trials that three human subjects made in the AXB psychophysical experiments. The dashed line represents chance level (50% of correct answer). *: significantly different from chance level using a chi-square test (fore detailed statistics, see text). (TIF) [file pbio.2001379.s005.tif]

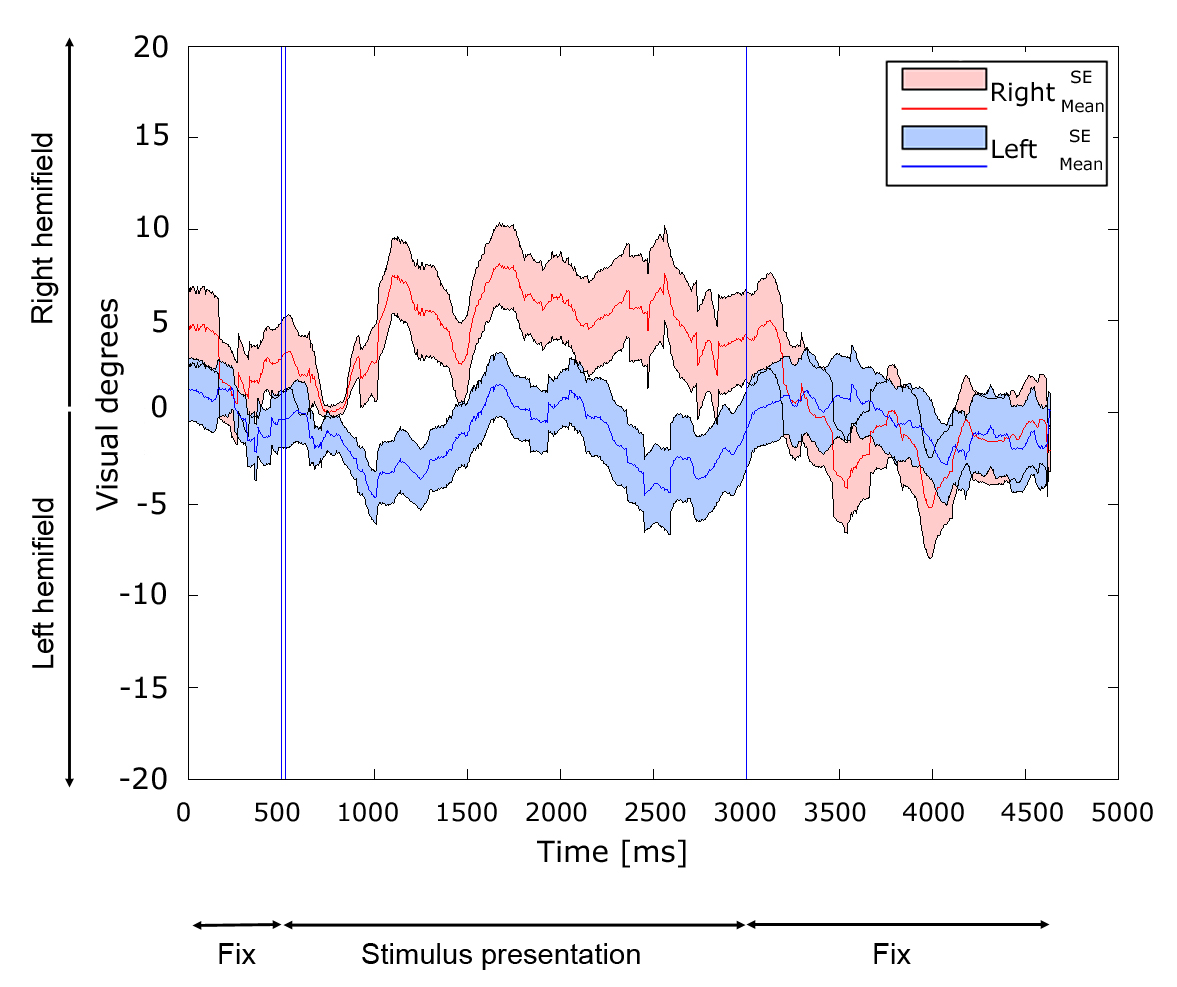

Supplement: S6 Fig — Eye movements when the subject was asked to not move his eyes (Fix, fixation spot on) and when subject heard the auditory stimulus while free to move his eyes (stimulus presentation, fixation spot off). Auditory stimuli were left (-80 or -40°) stationary stimuli (left) or right (40 or 80°) stationary stimuli (pink). (TIF) [file pbio.2001379.s006.tif]
